# Spoken language delay in toddlerhood predicts late childhood language outcomes in dyslexia: evidence from retrospective parent-report

**DOI:** 10.1007/s11881-026-00387-w

**Published:** 2026-07-31

**Authors:** Eleanor R. Palser, Nilgoun Bahar, Ellie Carpenter, Maya Bardorf, Margo Kersey, Rian Bogley, Sarah Inkelis, Pedro Pinheiro-Chagas, Boon-Lead Tee, Jessica DeLeon, Virginia E. Sturm, Zachary A. Miller, Maria Luisa Mandelli, Maria Luisa Gorno Tempini, Christa Pereira

**Affiliations:** 1 UCSF Dyslexia Center, Department of Neurology, University of California, San Francisco, San Francisco, CA 94143 USA; 2 Department of Psychology, Palo Alto University, Palo Alto, CA 94304 USA; 3 Department of Psychiatry and Behavioral Sciences, University of California, San Francisco, San Francisco, CA 94107 USA

**Keywords:** Literacy, Language delay, Language milestones, Reading, Specific learning disorder, Late-talking, Dyslexia

## Abstract

Some children are slower than average to acquire early spoken language milestones. Little is known about how this heterogeneity in expressive language impacts later childhood language and reading outcomes in children with dyslexia. We investigated oral language and reading differences in 63 children with dyslexia aged 8–12 years, comparing those with (35%) and without (65%) a prior history of delayed two-word phrase production (beyond 24 months of age). All children subsequently acquired fluent spoken language. Language outcome measures included standardized tests of current sound awareness, rapid picture naming, memory for words, and sentence repetition. Current single word and paragraph reading abilities were measured using standardized timed and untimed tests. Results indicated that both groups showed comparable single word and paragraph reading performance in middle childhood. Those with early spoken language delay had poorer sentence repetition and memory for words than those who used two-word phrases by 24-months. Our findings point to distinct language contributions to dyslexia in children with and without a history of early spoken language acquisition difficulties, underscoring the importance of considering early language milestones in this population.

**Supplementary Information:**

The online version contains supplementary material available at https://doi.org/10.1007/s11881-026-00387-w.

## Introduction

Developmental dyslexia (henceforth dyslexia) is a neurodevelopmental condition characterized by specific difficulties in reading, which cannot be better explained by differences in intelligence, other developmental factors, or inadequate instruction (Lyon et al., 2003). Dyslexia is most commonly diagnosed following the initiation of formal reading instruction in late childhood (i.e., between six and 12 years), a period that spans from the onset of formal education through to the beginning of adolescence. Prevalence estimates range between five and 17%, depending on the diagnostic criteria used (Di Folco et al., 2022). Research consistently highlights difficulties in phonological processing as central to dyslexia, both before and after the onset of reading instruction (Leppänen et al., 2012; Melby-Lervåg et al., 2012; Share, 2021; Thompson et al., 2015). There is also growing evidence of difficulties in other language domains, including delays in acquiring spoken (oral) language, which indicates variability in early language profiles among children who later develop dyslexia (Catts & Kamhi, 2005; Maxwell & Wallach, 1984). Not all children with dyslexia show early differences in spoken language, however, suggesting there may be common and distinct pathways to reading difficulties.

Independent of dyslexia, early spoken language represents an important early indicator of possible later academic achievement (Beitchman et al., 1996), behavioral problems (Beitchman et al., 1996; Benasich et al., 1993; Redmond & Rice, 1998), and social outcomes (Fujiki et al., 1996; Gertner et al., 1994). For instance, spoken language proficiency predicts speech-to-print conversion for beginning readers (Marks et al., 2019). Indeed, the association between early spoken language and later reading proficiency has been found as early as infancy, in the form of infant vocabulary (Duff et al., 2015), which suggests very early spoken language may impact later literacy. In children without dyslexia, early spoken language skills are important indicators of literacy and later academic outcomes (Hulme et al., 2015; Lyytinen et al., 2005; Snowling et al., 2000, 2016). In one study, five-year-old children at familial risk for dyslexia who also had spoken language delay at two years of age had poorer expressive language skills than children at risk for dyslexia who did not have spoken language delays (Lyytinen et al., 2005). A weak language foundation in childhood can hinder the development of skills necessary for fast and fluent reading (Botting et al., 2006; Snowling & Melby-Lervåg, 2016). This is supported by studies that have found a higher risk of reading problems in children with a history of spoken language difficulties (Snowling et al., 2000). Similarly, weaknesses in spoken language comprehension are a predictor of later reading difficulties in children with developmental language disorder (DLD, previously termed specific language impairment or SLI; Tallal et al., 1988), in part reflecting the influence of early phonological skills on the development of literacy (Hulme et al., 2015). Phonological challenges directly affect phonological awareness, a crucial skill for decoding the sound structure of written words (Bishop & Clarkson, 2003; Bortolini & Leonard, 2000). The relationship between early spoken language milestones and academic outcomes therefore merits further attention, specifically regarding contributions to later language and reading success.

The early language profiles of children who go on to develop dyslexia are variable but suggest there are possible early indicators of later reading challenges (Moll et al., 2013; Preston et al., 2010; Scarborough, 1990; Snowling et al., 2026; Spencer et al., 2014). The nature of these language difficulties appears to dynamically change across development, however. In a seminal study, Scarborough found that at age two and a half, toddlers who later met criteria for a reading disability showed reduced syntactic complexity and poorer pronunciation accuracy relative to their peers (Scarborough, 1990). At three years of age, weaknesses in receptive vocabulary and object-naming abilities emerged, and at five years, weaknesses in object-naming, phonemic awareness, and letter-sound knowledge were detectable (Scarborough, 1990). Since Scarborough’s study, there has been growing recognition of a link between early language milestones and subsequent reading difficulties (Moll et al., 2013; Snowling & Melby-Lervåg, 2016; Spencer et al., 2014). Further research is needed, however, to clarify which factors influence long-term literacy outcomes among children with dyslexia, particularly distinguishing between those who did or did not experience early language delays.

The most common causes of reading challenge in dyslexia are well established, including phonological challenges (Stanovich, 1988; Swan & Goswami, 1997), slow automatized naming (Wolff et al., 1990), and reduced verbal working memory capacity (Berninger et al., 2006), and continue to be investigated elsewhere. In this study, we focus on early risk factors, specifically early spoken language delay, that may have resolved at the onset of formal reading instruction yet continue to impact literacy development. Understanding these distal influences can not only clarify the literacy trajectories of late talkers but also inform early intervention plans.

The complex and overlapping nature of neurodevelopmental conditions mean that prospective studies of dyslexia may inadvertently include children from different diagnostic groups, introducing significant variability in literacy outcomes over time. Additionally, most prospective studies focus on children with a familial risk of dyslexia (e.g., Lyytinen et al., 2005), whose backgrounds may differ in important ways from those with dyslexia but no family history. Given heritability estimates of 40–60% (Fisher & DeFries, 2002; Raskind et al., 2013), it is essential to also include children without a family history to ensure findings are generalizable.

To address these issues, the present study examines whether parent-reported early spoken language milestones relate to reading and language profiles among children with dyslexia in late childhood. Such a retrospective approach allows us to rule out co-occurring neurodevelopmental conditions that become apparent with age, thereby reducing phenotypic heterogeneity. Specifically, we investigated how the language and reading profiles of these children varied based on their age of acquisition of phrase speech. Parents’ retrospective reports were used to form two groups of dyslexic children, comparing children with dyslexia who began using two-word phrases on time (i.e., prior to 24 months) versus those who were slow to reach this milestone (i.e., after 24 months), in line with previous research (e.g., Preston et al., 2010). Retrospective parent report is commonly used in studies of early language development and is considered a relatively reliable source of information on developmental history (Rescorla & Alley, 2001; Zubrick et al., 2007). We expected that children with dyslexia who had spoken language delay as toddlers would show more severe challenges with reading and lower performance on measures of oral language skills, including phonological awareness, rapid automized naming, and auditory short-term memory, than children with dyslexia without spoken language delay.

## Method

### Participant selection

The sample was derived from the cohort of research participants with digital records seen at the UCSF Dyslexia Center between 2015 and 2024. Participants underwent a comprehensive multidisciplinary neurobehavioral evaluation, including interviews with families and teachers, neurological examination with a neurologist, monitoring by a speech and language pathologist, and an academic and neuropsychological assessment with a trained evaluator under the supervision of a licensed clinical neuropsychologist. Diagnosis in all cases was specific learning disorder with impairment in reading.

### Record review

We undertook a chart review to identify children for whom we had digital records of their early language milestones (see Early Language Milestones section below). A total of 36 participants were excluded due to an absence of early language milestone record. This resulted in a sample of 98 participants. Participants were excluded if they had another condition known to impact cognition and language (e.g., fetal alcohol spectrum disorder). We excluded participants who had a co-occurring diagnosis of autism spectrum disorder (ASD; *n* = 2) as language delay in ASD is common and may have its own unique trajectory and pathophysiology (Andreo et al., 2024; Landry & Loveland, 1988). Previous diagnosis of an additional sporadic (i.e., assumed to appear at random) co-occurring neurodevelopmental condition was not an exclusion criterion, and the sample is therefore representative of the high co-occurrence of neurodevelopmental conditions within the wider population (August & Garfinkel, 1990; Gayán et al., 2005; Semrud-Clikeman et al., 1992; Willcutt & Pennington, 2000). See Supplementary Materials for a list of all co-occurring diagnoses in the sample.

### Assessment

In addition to the above criteria, inclusion in the current study required (1) at least one standardized reading test score below the 25th percentile (see ‘Reading Assessment’ section below), and (2) a standardized nonverbal reasoning score above the 9th percentile or a standardized receptive vocabulary score above the 25th percentile (see ‘Cognitive Assessment’ section below). These inclusion criteria were chosen to ensure participants had a specific learning disorder with impairment in reading without an intellectual developmental disability. The relatively liberal reading cut-off was chosen because many of the participants had received extensive reading remediation. From these, we excluded 33 participants who did not receive a consensus diagnosis of dyslexia (i.e., specific learning disorder in reading) by our multidisciplinary team.

Lastly, participants were reviewed for current or historical speech-related concerns, including speech sound distortions, motor speech difficulties, and disfluency, by our multidisciplinary team, which included a speech-language pathologist (SLP). Evidence of speech-related difficulties was identified through the SLP’s review of parent intake questionnaires, notes from language assessments completed by either the SLP or trained examiners, family and teacher conference notes, and other available clinical records for each child.

The final sample therefore included 63 dyslexic children ages eight to 12 years (60% male; 89% right-handed). Based on parent reports, participants fell into two groups diagnosed with dyslexia: one group of 22 children had dyslexia and spoken language delay and a second group had dyslexia but began using phrase speech at the typical time (*n* = 41). Among the 22 participants with dyslexia and a history of spoken language delay, six (27.3%) had a documented history of speech-related difficulties. These included mild-to-moderate phonological and articulation difficulties (*n* = 5) and one diagnosis of childhood apraxia of speech. Among the 41 with dyslexia without spoken language delay, nine (22%) had a history of speech-related concerns as per intake questionnaire and/or family conference notes. These included articulation difficulties (*n* = 5), motor speech difficulties suggestive of apraxic features during speech motor examination at our center (*n* = 2), and a reported history of stuttering (*n* = 2). All observed speech and fluency difficulties were mild or residual. In one child, speech sound distortions were judged to be temporarily associated with an orthodontic appliance.

The study protocol was approved by the Institutional Human Research Protection Program. Parents provided written informed consent, and participants provided verbal assent.

### Measures

#### Demographic measures

Parents were asked to report on participants’ age, sex, handedness, race, and household income. We also collected data on parental psychiatric history, family history of dyslexia, and maternal education, given known associations with early language development (Hoff-Ginsberg, 1998; Paulson et al., 2009). Household income was reported on a 15-point income band scale ranging from ‘less than $10,000’ to ‘over $500,000’. This scale represents a simplified version of the brackets used for standard household income reporting, including by the American Community Survey (United States Census Bureau, 2022), the main ongoing source of detailed income data in the United States. Maternal education was reported as the number of years of completed education.

The sample was predominantly white (75%), followed by multiracial/multiethnic (32%), Asian/Pacific Islander (10%), Latino (8%), and African American/Black (2%). Five percent of parents declined to report their child’s race. The annual household income of the sample ranged from the $30,000–39,999 band to over $500,000 with a median income band of $250,000–299,999, indicating a relatively high socio-economic status, relative to the median household income of the local area, which is $137,000–141,000 (United States Census Bureau, 2024). Sixteen percent of parents declined to report their household income. Demographic characteristics of the sample are reported in Table 1.

**Table 1:** Group-wise descriptive statistics for demographic characteristics, reading, and language performance. For standardized test scores, we provide the median, range, 25th, and 75th percentile scores

Measure	Dyslexia with spoken language delay (*n* = 22)	Dyslexia without spoken language delay (*n* = 41)
Age *M* (*SD*)^1^	10.2 (2.1)	10.6 (2.3)
Household income Range, Median^2^	$30,000–39,999 -500,000 + , $200,000–299,999	$80,000–500,000 + ,$300,000–399,999
Maternal education Median^3^	Graduate degree	College degree
Family history of dyslexia %^4^	23%	36%
Parental psychiatric history %^4^	23%	10%
Matrix reasoning Range, Median (25th,75th)^5^	3–99, 56.5 (27,82)	1–98, 48 (27,75)
Symbol search Range, Median (25th,75th)^6^	1–84, 25 (5,56)	1–95, 37 (16,50)
Receptive vocabulary Range, Median (25th,75th)^7^	10–99, 61 (42,79)	16–99, 77 (47,87)
Expressive vocabulary Range, Median (25th,75th)^8^	2–99, 36.5 (14,58)	4–96, 49.5 (13.5,70)
Sight word efficiency Range, Median (25th,75th)^9^	< 1–70, 8.5 (2,18)	< 1–55, 5 (1,16)
Phonemic decoding efficiency Range, Median (25th,75th)^9^	< 1–53, 4 (1,13)	< 1–58, 8.5 (2,19)
Paragraph reading accuracy Range, Median (25th,75th)^10^	< 1–84, 5 (2,9)	< 1–63, 5 (2,16)
Paragraph reading rate Range, Median (25th,75th)^10^	< 1–63,9 (2,25)	< 1–37, 9 (2,25)
Paragraph reading fluency Range, Median (25th,75th)^10^	1–63, 5 (2,9)	1–50, 7 (2,16)
Paragraph reading comprehension Range, Median (25th,75th)^10^	1–84, 9 (2,16)	1–75, 16 (9,25)
Sound awareness Range, Median (25th,75th)^11^	1–95, 25.5 (9,42)	3–82, 43.5 (14,63)
Rapid picture naming Range, Median (25th,75th)^11^	1–47, 21.5 (10,37)	1–81, 35.5 (18,53)
Sentence repetition Range, Median (25th,75th)^11^	4–88, 18.5 (13,44)	3–94, 46 (23,63)
Memory for words Median (25th,75th)^11^	1–58, 14.5 (6,47)	4–99, 32.5 (19,45)

1) Age is reported in years. 2) Household income was self-reported in U.S. dollars. 3) Maternal education was self-reported. 4) Family history of dyslexia and parental psychiatric history reflect the percentage of children in each group with a positive history. 5) Matrix Reasoning was measured using the Wechsler Abbreviated Scale of Intelligence (WASI; Wechsler, 1999). 6) Symbol Search was measured using the Wechsler Intelligence Scale for Children – Integrated – Fourth Edition (WISC-IV Integrated; Wechsler et al., 2004). 7) Receptive vocabulary was measured using the Receptive One-Word Picture Vocabulary Test – Fourth Edition (ROWPVT-4; Martin & Brownell, 2011). 8) Expressive Vocabulary was measured using the Woodcock-Johnson, Fourth Edition Tests of Oral Language (Schrank et al., 2014), Oral Vocabulary subtest. 9) Sight word efficiency and phonemic decoding efficiency were measured using the Test of One-Word Reading Efficiency – Second Edition (TOWRE-2, Torgesen et al., 2012). 10) Paragraph reading accuracy, rate, fluency, and comprehension were measured using the Gray Oral Reading Test – Fifth Edition (GORT-5; Wiederholt & Bryant, 2012). 11) Sound awareness, rapid picture naming, sentence repetition, and memory for words were measured using the Woodcock-Johnson, Fourth Edition, Tests of Oral Language (Schrank et al., 2014)

#### Early language milestones

Parents of participants were asked to retrospectively report the age (in months) at which their child said their first word and first began using two-word phrases. Participants were categorized as having experienced an early spoken language delay if they first used two-word phrases later than 24 months (Paul, 1991). This index of early spoken language delay was chosen because forming early word combinations has been found to be a highly reliable estimate for late language emergence (Zubrich et al., 2007), correlate with long-term functional and structural differences in brain activity in language relevant regions (Dynak et al., 2021; Preston et al., 2010), and relate strongly to later language and literacy skills (Lyytinen et al., 2001; Scarborough & Dobrich, 1990). Converging evidence in the present dataset indicated that the age at which children began using two-word phrases was highly correlated with the age at which they spoke their first word, *r*_*s*_ (56) = .48, *p* < .001.

#### Cognitive assessment

All participants completed Matrix Reasoning, a test of nonverbal reasoning from the Wechsler Abbreviated Scale of Intelligence (WASI; Wechsler, 1999), which requires selecting a visual pattern from a set of options that represents the missing piece in a set of visually presented pieces. Processing speed was measured using the Symbol Search subtest from the Wechsler Intelligence Scale for Children – Integrated – Fourth Edition (WISC-IV Integrated; Wechsler et al., 2004). This subtest requires participants to rapidly determine whether a target symbol is present in a row of visually presented symbols.

#### Language assessment

All participants completed the Receptive One-Word Picture Vocabulary Test–Fourth Edition (ROWPVT-4; Martin & Brownell, 2011), a measure of receptive vocabulary in which participants select the picture corresponding to a word presented orally by the examiner. Participants also completed the following subtests from the *Woodcock-Johnson IV Tests of Oral Language* (Schrank et al., 2014): Oral Vocabulary**,** which assesses expressive vocabulary through the production of synonyms and antonyms; Sentence Repetition and Memory for Words, which assess auditory short-term memory; Sound Awareness, including the Rhyming and Deletion subtests, which measure phonological awareness by requiring participants to generate a rhyming word or delete a specified syllable from an orally presented word; and Rapid Picture Naming, which assesses lexical retrieval speed by asking participants to name as many pictured objects as possible within a two-minute time limit.

#### Reading assessment

All participants completed the Test of One-Word Reading Efficiency – Second Edition (TOWRE-2, Torgesen et al., 2012) to assess their timed single-word reading abilities, which provides scores for both sight word efficiency and phonemic decoding efficiency. Participants completed the Gray Oral Reading Test – Fifth Edition (GORT-5; Wiederholt & Bryant, 2012) to assess their oral paragraph reading, which provides scores for accuracy, rate, and comprehension. Fluency scores were additionally calculated based on reading accuracy and rate.

### Data analysis

Data analyses were conducted in R Studio (R version 4.2.0; R Core Team, 2022). Two-tailed tests were used throughout. First, we examined whether the groups were matched on the frequency of co-occurring diagnoses and on variables that may influence language development (i.e., age, sex, household income, parental history of psychiatric problems [binarized as positive or negative for endorsement of any psychiatric diagnosis], nonverbal reasoning, processing speed, receptive vocabulary, and expressive vocabulary). Fischer-Irwin tests were used to compare the frequency of co-occurring diagnoses between the two groups. A Chi-squared test was used to compare the groups on sex, household income, family history of dyslexia, and parental history of psychiatric problems. Multiple linear regression tests were used to compare the groups on nonverbal reasoning , processing speed, receptive vocabulary, and expressive vocabulary. Raw scores for cognitive and language measures were used in these analyses, and as such, we controlled for age and sex. Second, we used a *t*-test to confirm that the age of acquisition of two-word phrases was significantly older in participants with spoken language delay than in children without spoken language delay.

Third, we investigated group differences in current language and reading abilities. We used multiple linear regression tests, entering raw scores as the dependent variable, and controlled for age, sex, and household income. Language outcomes included sound awareness, sentence repetition, memory for words, and rapid picture naming. Reading outcomes included single sight word efficiency and phonemic decoding efficiency, as well as paragraph reading fluency, rate, accuracy, and comprehension. For measures of language and reading, we report the proportion of children in each group whose current performance falls in the below average range (i.e., below the ninth percentile) using the consensus benchmarks established by American Academy of Clinical Neuropsychology (AACN; Guilmette et al., 2020) and report percentile scores in tables and figures, for ease of interpretation.

## Results

### Group matching

Frequencies of co-occurring diagnoses were similar between the two groups (see Supplementary Table 1). The two groups were also matched at the group-level on sex (*χ2* = 0.87, *p* = .350), age (*t*(46) = 0.63, *p* = .532), nonverbal reasoning (*Β* = 1.09, *t* = 0.75, *p* = .456), processing speed *(Β* = 1.51, *t* = 0.75, *p* = .455), receptive vocabulary (*Β* = 7.06, *t* = 1.76, *p* = .084), and expressive vocabulary (*Β* = 1.92, *t* = 1.48, *p* = .145). Rates of both family history of dyslexia (*χ2* = 0.12, *p* = .735) and parental history of psychiatric problems (*χ2* = 2.21, *p* = .137) were also similar between the groups. The children with dyslexia without spoken language delay had greater representation at the highest income stratum than the children with dyslexia with spoken language delay (*χ2* = 11.65, *p* < .001). We therefore controlled for household income in our analyses of language and reading outcomes. Children with dyslexia with spoken language delay were significantly older in acquiring two-word phrases than the children with dyslexia without spoken language delay (per the group selection method): *t*(25) = 9.49, *p* < .001). See Table 1 for descriptive statistics for both groups.

### Reading abilities

There were no differences between the groups in sight word efficiency (*Β* = 0.54, *t* = 0.12, *p* = .904), or phonemic decoding efficiency (*Β* = 1.83, *t* = 0.61, *p* = .543), when reading single words. Fifty percent of the children with dyslexia and spoken language delay and 56% of the children with dyslexia without spoken language delay scored below the average range in sight word efficiency. Sixty eight percent of the children with dyslexia and spoken language delay and 49% of the children with dyslexia without spoken language delay scored below the average range in phonemic decoding efficiency. There were also no differences between the groups in paragraph reading fluency (*Β* = 0.89, *t* = 0.19, *p* = .851), rate (*Β* = 1.00, *t* = 0.41, *p* = .683), accuracy (*Β* = 1.90, *t* = 0.70, *p* = .486), or comprehension (*Β* = 3.69, *t* = 1.49, *p* = .144). Descriptively, a greater proportion of children with dyslexia and early spoken language delay performed below the average range than children with dyslexia and no early spoken language delay in reading fluency (68% versus 49%, respectively), accuracy (73% versus 51%, respectively), and comprehension (36% versus 22%, respectively). By contrast, a greater proportion of the children with dyslexia and no early spoken language delay performed below the average range on reading rate than children with both dyslexia and early spoken language delay (27% versus 37%, respectively).

### Language abilities

**Fig. 1:**
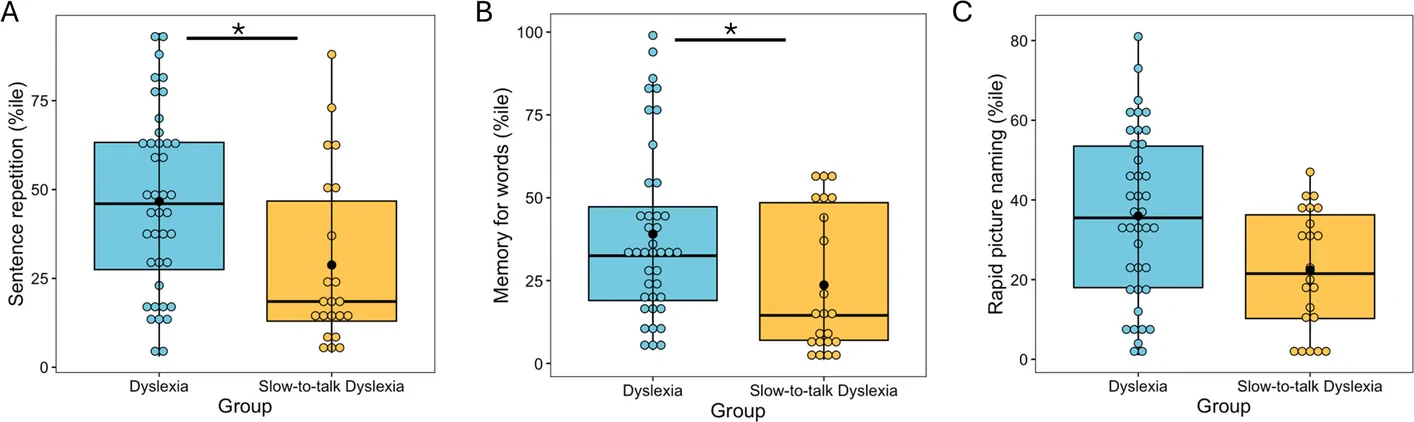
Children with dyslexia and early spoken language delay had poorer sentence repetition and memory for words than children with dyslexia without early spoken language delay. Boxplots indicate first quartile, median, and third quartile; whiskers show first and third quartile ± 1.5*interquartile range. Asterisk indicates significance at *p* < .05

Children with dyslexia and spoken language delay had poorer sentence repetition (*Β* = 2.76, *t* = 2.71, *p* = .001; see Fig. 1A), and poorer memory for words (*Β* = 1.95, *t* = 2.24, *p* = .030; see Fig. 1B), than children with dyslexia without spoken language delay. Sentence repetition ability was in the below average range in 23% of the children with dyslexia and spoken language delay and 5% of the children with dyslexia without spoken language delay. Memory for words performance was in the below average range in 36% of the children with dyslexia and spoken language delay and 7% of the children with dyslexia without spoken language delay. There was also a trend towards poorer rapid picture naming in the children with dyslexia and spoken language delay (*Β* = 8.34, *t* = 1.96, *p* = .057), with 23% of the children with dyslexia and spoken language delay scoring in the below average range, relative to 17% of the children with dyslexia and no spoken language delay. There were no differences between the groups in sound awareness (*Β* = 2.64, *t* = 1.47, *p* = .147), with 23% of the children with dyslexia and spoken language delay scoring in the below average range, and 15% of the children with dyslexia and no spoken language delay.

## Discussion

The present study examined the influence of early spoken language milestones, specifically the onset of phrase speech as retrospectively reported by parents, on late childhood language and literacy outcomes in children with dyslexia. By comparing language and literacy outcomes between dyslexic children identified as having early spoken language delay versus those who produced two-word phrases on-time, we sought to better understand the long-term developmental trajectories associated with early spoken language delays. Our findings indicate that individual differences in spoken language development evident by age two are associated with language outcomes during late childhood among children diagnosed with dyslexia. Specifically, performance on tests of sentence repetition and memory for words was notably poorer in dyslexic children who experienced early spoken language delay compared to those who used phrase speech on time. These outcomes underscore the persistence of residual language difficulties into late childhood for a subset of dyslexic children who demonstrated delayed language milestones as toddlers.

Differences in late childhood language skills between children with and without prior difficulties in meeting their early language milestones suggest that the underlying mechanisms of reading difficulties in these children may differ; the reading challenges in the group with spoken language delay may be secondary to or co-occurring with broader language vulnerabilities. Indeed, previous research has shown that early spoken language delay and familial risk for dyslexia have independent effects on language skills in late childhood, and that those with early spoken language delay have reduced activation in a network of regions including the thalamus and putamen bilaterally, left insula, and left superior temporal gyrus during exposure to speech and print in late childhood, compared to children who talked on time (Preston et al., 2010). Structurally, they also have reduced grey matter volume in the left middle temporal cortex (Raschle et al., 2017) and reduced cortical thickness in the left posterior cingulate gyrus and the right superior temporal gyrus (Dynak et al., 2021). These findings have important implications for how clinicians and educators intervene and support children’s emerging literacy. It also highlights the importance of early language screening with the objective of minimizing or even preventing reading difficulties secondary to a weak language foundation in the pre-school years (Catts & Hogan, 2021).

Perhaps consistent with frameworks that posit that phonological weaknesses represent the most common core vulnerability in dyslexia (Pennington, 1995; Stanovich, 1988; Swan & Goswami, 1997), both groups of children showed comparable sound awareness performance. Despite variability in scores within the groups, the most notable performance gap between these two groups emerged on a test of sentence repetition. This parallels a previous investigation of 13-year-old children with early spoken language delay, who generally showed similar academic and language performance, but challenges in verbal memory, as well as grammar and vocabulary, relative to age-matched peers without early spoken language delay (Rescorla, 2000). In Rescorla’s (2000) cohort, the adolescents with prior early spoken language delay appeared to perform most poorly on tasks that required mental manipulation of verbal information, such as repeating ambiguous sentences. Accurately repeating a sentence requires the child to regenerate a grammatically correct sentence, which in part, depends on the strength of their prior language foundation, as well as the ability to maintain a conceptual representation in mind. As such, sentence repetition is a multifaceted task that taxes multiple aspects of language simultaneously (Klem et al., 2015; Potter, 2012). Prior studies show that this test represents an independent dimension of linguistic prowess, loading strongly onto a unidimensional language factor (Klem et al., 2015; Tomblin & Zhang, 2006). Children with dyslexia but without early spoken language delay may be able to compensate for weak phonological skills using other higher-order language abilities (Goulandris et al., 2000). The variability observed even amongst the children with spoken language delay, ranging from below average to high average, may reflect differences in broader language skills that may be used to compensate for phonological weaknesses. Given the multidimensional nature of this task and its sensitivity to linguistic competence beyond the phonological short-term memory limitations commonly associated with dyslexia, our findings point toward deeper underlying language challenges in dyslexic children with early spoken language delay.

The test of sentence repetition is widely regarded as a highly sensitive measure for identifying children with DLD, capturing even subtle language difficulties that may not manifest in other linguistic assessments (Conti-Ramsden et al., 2001; Stothard et al., 1998). Interestingly, many children who meet the diagnostic criteria for DLD are not identified until they reach school age, when reading difficulties become evident (Botting et al., 2006; Catts et al., 2005). This underscores the subtle nature of language impairments that might go unnoticed until the onset of formal schooling. The frequent overlap between language and reading difficulties raises questions about the relationship between DLD and dyslexia, and the extent to which the two constitute separable conditions (Snowling et al., 2026). Like DLD, dyslexia is a persistent neurodevelopmental condition with a strong genetic basis (Bishop & Snowling, 2004). Neuroimaging studies have reported overlapping functional and structural differences in several cortical areas, including the ventral occipitotemporal region as well as the inferior frontal gyrus in both conditions (Bahar et al., 2024; Dynak et al., 2021; Frye et al., 2010; Kronbichler & Kronbichler, 2018; Paulesu et al., 2014; Raschle et al., 2017). Both conditions are dimensional, varying in severity, with no clear boundaries distinguishing one from the other. DLD and dyslexia are therefore currently understood as distinct but closely related conditions that commonly co-occur (Bishop et al., 2009; Catts et al., 2005; Ramus et al., 2013; Snowling et al., 2019, 2026).

Considering the established similarities between dyslexia and DLD, it is plausible that our cohort of dyslexic children with early spoken language delay represent cases of co-occurring dyslexia and language impairment (Adlof & Hogan, 2018). Children in this category are often at risk of difficulties with decoding as well as understanding text, which places them at an elevated risk for the most severe reading comprehension problems (Snowling et al., 2019). Although not statistically significant, our cohort of dyslexic children with spoken language delay did descriptively have lower mean scores in reading comprehension compared to their non-delayed peers with dyslexia. Although a more comprehensive test battery would be required to assess the presence of DLD in our sample, it is notable that only one participant in our sample had received a prior diagnosis of DLD, which speaks to the hidden nature of language difficulties, showing that not all children who are late talkers will reach clinical attention. Our results suggest that for some children this may hold true, with some showing early delays in acquiring spoken language, followed by subsequent literacy challenges and continuing difficulties with word retrieval. Other children in our sample, however, never apparently demonstrated spoken language difficulties, and have a comparably isolated challenge with reading.

Children with dyslexia who experienced early spoken language delay also had poorer memory for words in late childhood than their peers with dyslexia who spoke on time. Memory for words places similar demands on the ability to temporarily hold linguistic information in awareness to sentence repetition, pointing to a shared underlying weakness in auditory short-term memory. Although it was just below the threshold of statistical significance, there was also evidence of poorer rapid picture naming in children who had early spoken language delay, suggesting that retrieving lexical information quickly, as well as its short-term storage, may be challenging for this sub-population. It is possible that remediation efforts may have more extensively targeted phonological awareness than auditory short-term memory and rapid picture naming. Another possibility is that the children with spoken language delay may have always been weaker in auditory short-term memory and rapid lexical retrieval than the on-time talkers. Further studies of longitudinal development that account for the type of intervention received would help to address this question. Single-word and paragraph reading abilities were comparable across the groups, suggesting that the more severe auditory short-term memory and rapid lexical retrieval difficulties seen in the children with spoken language delay may persist even when their reading looks similar to other poor readers. This is in line with previous work that has shown that current language skills mediate the association between early language acquisition and current reading skills in children with dyslexia (Price et al., 2022). Children with dyslexia who also had early spoken language delay may benefit from additional support in the form of compensatory strategies for their weaknesses in auditory short-term memory. Moreover, following up on what aspects of sentence repetition children with concurrent reading and language difficulties struggle with may be a valuable clinical tool to tease apart the underlying processes that pose challenges to children with dyslexia (Moll et al., 2013; Nag et al., 2013).

Given the variability associated with the dyslexia phenotype explored here, it is likely that in many cases the reading difficulties overlap with or might be caused primarily by oral language difficulties in early childhood. The causal relations between early delays in acquiring phrase speech and later language difficulties such as poor sentence repetition are likely indirect and multifaceted. Delays in achieving early language milestones may limit young children’s participation in activities that promote language development and scaffold literacy, including early print exposure, joint book reading, parent–child conversations, and other language-dependent educational tasks. This reduced participation could in turn impact the acquisition of skills and metalinguistic awareness necessary for acquiring letter-sound correspondence and word retrieval rules. The flip side of this argument holds as well; it is likely that in cases of dyslexic children without a history of delayed language, a dominant cognitive factor related to phonological processing contributes to children’s reading difficulties, which in turn limits further engagement with language and literacy prompting activities required for learning complex vocabulary and grammar (Korochkina et al., 2024; Nation et al., 2022).

These results suggest there may be benefits to deeper phenotyping in dyslexia. Children with similar academic profiles (i.e., challenges with reading) may mask different underlying language profiles and developmental trajectories. The children included in this study, all diagnosed with dyslexia and showing similar reading performance at this stage of late childhood, may diverge in adolescence if underlying oral language vulnerabilities are not remediated. Phenotyping therefore seeks to delineate the underlying mechanisms the contribute to the observed academic difficulties and inform the intervention approach. There have long been recommendations to adopt a subtyping approach in dyslexia, which classifies poor readers based on their underlying cognitive or language profiles (Bishop & Snowling, 2004; Chalmpe & Vlachos, 2025; Frith, 1999; Snowling, 2000). Taxonomies that classify poor readers based on their most prominent current area of weakness often leave some children with dyslexia unclassified and indicate a large amount of overlap between subtypes. These children may receive interventions that are poorly tailored to their developmental trajectory and underlying cognitive weaknesses and subsequently struggle to make gains in reading. A multidimensional model that considers performance on several underlying cognitive processes as opposed to discrete categories has since been advocated for (e.g., Bishop & Snowling, 2004). The current results suggest that early language milestones at age two may also be an important developmental signal that could improve the classification of dyslexia subtypes.

Finally, the observed poorer performance of late-talking dyslexic children on two predictors of reading difficulty (namely auditory short-term memory and rapid naming) is reminiscent of the double-deficit hypothesis proposed by Wolf and Bowers (1999). This theory suggests that those with the most severe difficulties in reading have both weaknesses in phonological processing and rapid automized naming. Clarifying these cognitive factors further may elucidate the neurobiological and genetic foundations of dyslexia, ultimately aiding in the development of more personalized and effective screening, assessment, and intervention strategies aimed at improving long-term outcomes for affected children. Specifically, it is possible that challenges in phonological processing and rapid automized naming are present in these children prior to late childhood and represent an early risk profile that is detectable prior to the onset of reading instruction. Early identification of children with spoken language delay and assessment of both phonological processing and rapid automized naming could represent an opportunity to prompt early intervention that may mitigate vulnerability to later emerging reading disability.

### Limitations and future directions

#### Retrospective report

In this study, we defined early spoken language delay as not producing a two-word phrase prior to 24 months (Paul, 1991), measured via retrospective parent-report. Other researchers have used alternative criteria, including having a vocabulary of less than 50 words by age two (Irwin et al., 2002; Poll & Miller, 2013), as well as combinations of these two variables (Rice et al., 2008). Future work is required to confirm that the results generalize to other features of early language delay and would be strengthened by the inclusion of multiple indicators. It is possible that some of the children with dyslexia who spoke their first two-word phrase prior to 24 months had, nonetheless, acquired less than 50 unique words and would be categorized as language delayed by other researchers. This may explain some of the variability in performance we observed in this group in late childhood. Longitudinal designs are also needed to mitigate some of the downsides of the retrospective report approach that we used here, including erroneous or biased recall (Majnemer & Rosenblatt, 1994), and confirm the present findings. A chart review approach, that verifies the veracity of parents’ recall through the examination of medical records, could represent a less time-consuming next step in replicating the present findings in a larger sample.

A further limitation is that speech-related difficulties in our cohort were documented through a clinical review rather than standardized speech assessment. Although our multidisciplinary review incorporated parent intake questionnaires, clinician and speech-language pathologist notes from language assessments, family and teacher conference notes, and available clinical reports, we did not administer standardized measures of speech sound production to systematically characterize these features. Future studies should incorporate formal speech assessments to more precisely define the nature and severity of speech-related difficulties and determine whether specific speech profiles are differentially associated with the two groups of dyslexic children investigated.

Our sample was comprised of children with dyslexia who had received extensive remediation and continued to show difficulties in reading. While this ensures that our research sample of children with dyslexia is limited to children who have a “true” specific learning disorder for reading and are not otherwise poor readers due to myriad other factors, including limited exposure or inattention, this may also have resulted in an excessively positive impression of language and reading in children with both early speech delay and dyslexia. The two cohorts, for example, showed comparable single word and paragraph reading abilities. Furthermore, it remains to be seen how our findings generalize to children who have not had access to specialized schooling and remediation. Future longitudinal studies may wish to examine rates of change in language and reading between children with dyslexia with and without early speech delay in more diverse samples.

This study did not include children with early language delay who did not go on to develop dyslexia. Future research studies should recruit children who are now typical readers but who experienced a language delay as toddlers, to investigate the factors that contributed to the development of typical reading in the face of early language delay, and better understand the features of early language that do not contribute to reading, as well as protective factors that temper against risk for dyslexia.

## Conclusions

In sum, this preliminary investigation using retrospective parent-report suggests that early spoken language delay may account for some of the variability observed in both research and clinical samples of children with dyslexia. Based on the findings reported, we recommend that early developmental language milestones be routinely assessed and included as potentially informative datapoints in future research designs and, if replicated, dyslexia evaluations. Preschool language difficulties could serve as an early indicator for clinicians, teachers, and parents of the need for additional, child-specific support and age-appropriate therapeutic strategies that support language and memory could be employed. Examples include coaching parents on strategies to follow the child’s lead, increasing caregiver responsiveness, and modeling language with expansions. Integrating visual aids, breaking information into manageable segments (chunking), employing mnemonic devices, and fostering active listening skills are also a few memory supporting strategies to consider for this population. Future research should explore whether early intervention in this vein would impact the course of children at-risk for dyslexia due to early language delays. 

## Supplementary Information

Below is the link to the electronic supplementary material.Supplementary file1 (DOCX 15 KB)

## Data Availability

Data requests can be submitted thought the UCSF Memory and Aging Center Resource Request form: http://memory.ucsf.edu/resources/data. Academic, not-for-profit investigators with Institutional Review Board approval from the UCSF Human Research Protection Program (HRPP) can request data for research studies. The UCSF HRPP will not review the application until the UCSF Memory and Aging Center Executive Committee has signed off on the proposal and consent form. Data are not publicly available because they contain information that could compromise the privacy of the participants.
