# Supplementary material for: Spoken language delay in toddlerhood predicts late childhood language outcomes in dyslexia: evidence from retrospective parent-report
Source: Ann Dyslexia. 2026 Jul 31;76(2):329–47. doi: 10.1007/s11881-026-00387-w (PMC13529838; doi:10.1007/s11881-026-00387-w)
Supplement: Supplementary file 1 [file 11881_2026_387_MOESM1_ESM.docx]

**Spoken language delay in toddlerhood predicts late childhood language outcomes in dyslexia: evidence from retrospective parent-report**

**Supplementary Materials**

**Supplementary Table 1:**

| **Co-occurring diagnoses** | **Slow-to-talk Dyslexia (*n* = 22)** | **Dyslexia (*n* = 41)** | **Comparison** |
| --- | --- | --- | --- |
| **ADHD** | *n* = 8, 36% | *n* = 15, 36.5% | Fischer-Irwin statistic = 1.00, *p* > .05. |
| **Dyscalculia** | *n* = 5, 23% | *n* = 8, 19.5% | Fischer-Irwin statistic = 0.75, *p* > .05. |
| **Dysgraphia** | *n* = 5, 23% | *n* = 11, 27% | Fischer-Irwin statistic = 0.77, *p* > .05. |
| **Developmental coordination disorder** | *n* = 2, 9% | *n* = 2, 5% | Fischer-Irwin statistic = 0.61, *p* > .05. |
| **Anxiety** | *n* = 2, 9% | *n* = 4, 10% | Fischer-Irwin statistic = 1.00, *p* > .05. |
| **Apraxia of speech** | *n* = 1, 4.5% | *n* = 0, 0% | Fischer-Irwin statistic = 0.35, *p* > .05. |
| **Specific language impairment** | *n* = 1, 4.5% | *n* = 0, 0% | Fischer-Irwin statistic = 0.35, *p* > .05. |
